# Supplementary material for: Investigating the oncogenic role of aberrant EZH2 in hepatoblastoma
Source: Sci Rep. 2026 Feb 6;16:7563. doi: 10.1038/s41598-026-38038-0 (PMC12932671; doi:10.1038/s41598-026-38038-0)
Supplement: Supplementary file 1 — Supplementary Information. [file 41598_2026_38038_MOESM1_ESM.pdf]

Supplemental Figure S1

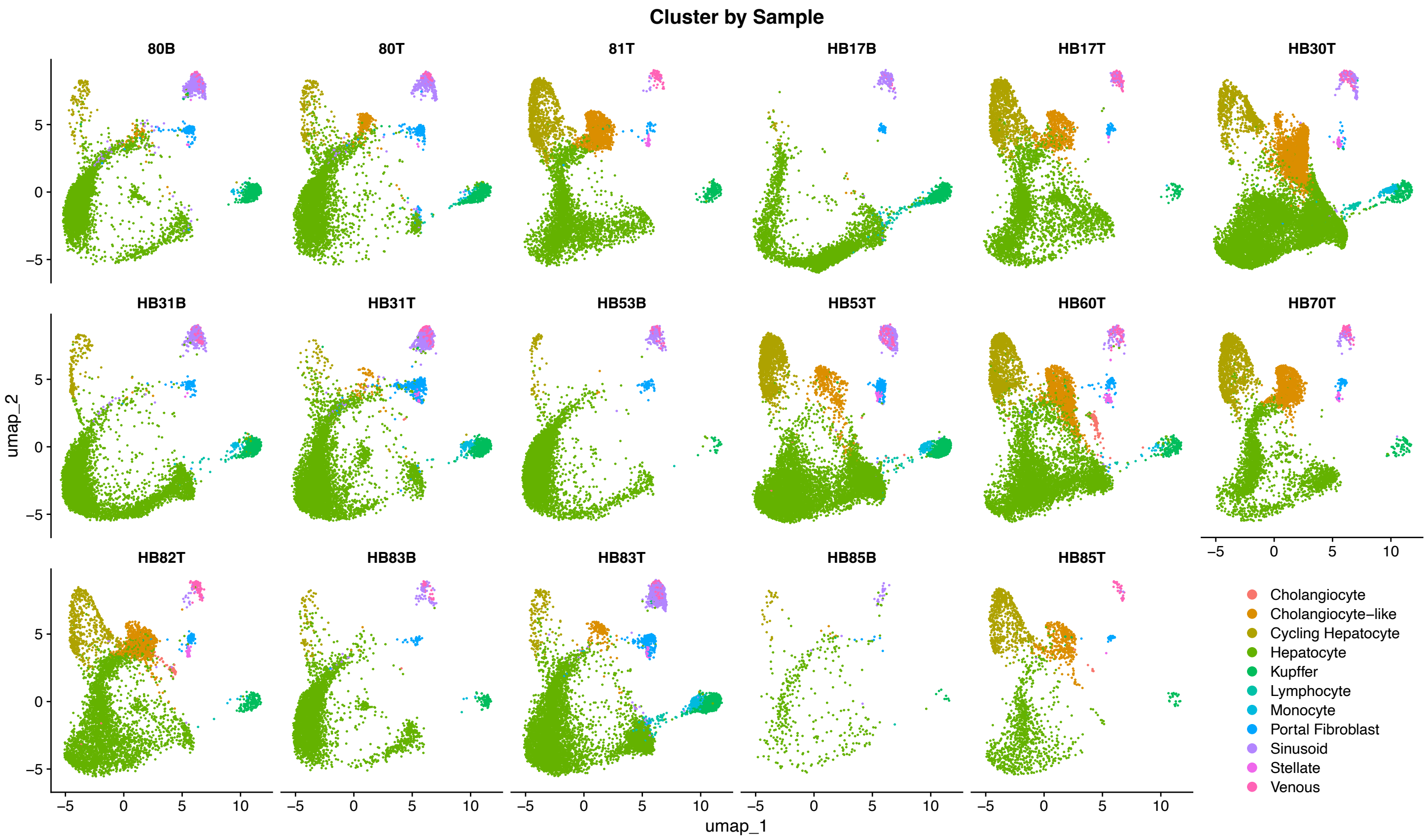

Supplemental Table 1- Marker Genes						
**Hepatocyte**	**Cholangiocyte**	**Cholangiocyte-like**	**Immune**	**Endothelial**	**Mesenchymal**	**Cycling**
GLUL	SPP1	KRT19	CD8A	STAB1	PRKG1	MKI67
CYP2E1	KRT7	JARID2	CD4	STAB2	DPT	EZH2
ALB	EPCAM	NKD1	PTPRC	PDPN	FBLN1	SUZ12
ASL			CD3G	CLEC14A	FBLN2	EED
ASS1			CD68	SELP	VCAN	AURKB
GLS2			SLC8A1	GJA5	MYH11	ASPM
ARG1			AOAH	FLRT2	MSLN	TOP2A
CYP2A1			VCAN			HELLS
OAT			S100A8			
AFP			CD14			
STAT3			MS4A1			

Supplemental Table 2 - Patient Sample Demographics

Patient ID	Description	Gender	Age at sample collection (years)	Pretext stage	Histology	Predominant histologic subtype	Neoadjuvant Chemotherapy prior to acquisition
17	HB	F	5.21	IV	fetal	fetal	Y
18	HB	M	2.21	III	mixed epithelial, fetal, embryonal, minor mesenchymal	mixed	Y
21	HB	M	2.96	IV	Predominant pattern pleomorphic; embryonal; HCC-like	embryonal	Y
23	HB	F	1.51	III	variable histology, no classic fetal/embryonal		Y
24	HB	F	3.81	IV	HCC-like		Y
25	HB	M	2.02	IV	cartilage		Y
27	HB	F	1.73	IV	Crowded fetal, rare multinucleated and pleomorphic, blastemal, pseudoacinar	mixed	Y
28	HB	F	1.58	III	bone, cartilage		Y
29	HB	M	1.88	IV	Metastatic HB, embryonal, undifferentiated morphology	embryonal	Y
30	HB	M	3.40	IV	Crowded fetal, embryonal, blastemal, rare pleomorphic	transition	Y
31	HB	M	2.19	II	mixed epithelial (fetal and embryonal) and mesenchymal without teratoid features	mixed	Y
38	HB	M	2.90	IV	Crowded fetal, blastemal, cholangioblastic	fetal	Y
40	HCC	M	27.08	I	Well-differentiated HCC	HCC	N
41	HB	M	2.06	IV	Crowded fetal, embryonal, blastemal	mixed	Y
42	HB	M	3.09	IV	HCC-like (pleomorphic, steatosis)		Y
43	HB	M	1.51	I	epithelial type, fetal, blastemal	fetal	N
45	HB	M	7.98	III	Epithelial type, macrotrabecular, embryonal, fetal	mixed	Y
46	HB	F	2.64	II	mixed fetal and embryonal, mesenchymal type without teratoid features	mixed	Y
47	HB	M	3.59	III	epithelial type, fetal (crowded), embryonal, blastemal, pleomorphic	mixed	Y
48	HCN-NOS	M	11.90	IV	fetal-like, macro, mild pleomorphism	HCN-NOS	Y
49	HB	M	0.38	III	epithelial type, fetal, embryonal (minor amount), blastemal, pleomorphic	fetal	Y
50	HB	F	0.64	II	Crowded fetal, blastemal, osteoid	fetal	Y
52	HCN-NOS	M	12.15	IV	HCN-NOS	HCN-NOS	Y
53	HB	F	2.20	IV	Crowded fetal, embryonal, blastemal	embryonal	Y
60	HB	M	3.57	II	Epithelial type, predominantly embryonal with fetal and blastemal components	embryonal	Y
62	HB				Metastatic HBL, mesenchymal, osteoid	mesenchymal	Y
64	HB	M	1.63	IV	Metastatic HBL, mesenchymal, osteoid	mesenchymal	Y
66	HB	F	3.94	III	epithelial type, mixed fetal and embryonal with focal macrotrabecular and minor blastemal	mixed	Y

67	HB	F	5.04	II	epithelial type, mixed embryonal and fetal blastemal, pleomorphic	mixed	Y
69	HB	F	0.84	III	Crowded fetal	fetal	Y
70	HB	M	4.00	II	Metastatic HB, epithelial type, predominantly embryonal with crowded-fetal and minimal blastemal component	embryonal	Y
71	HCN-NOS	M	7.56	IV	HCN-NOS	HCN-NOS	Y
73	HB	M	1.19	IV	epithelial, predominantly fetal, only small embryonal	fetal	Y
74	HB	F	3.35	IV	Crowded fetal, embryonal, blastemal, mild pleomorphic	embryonal	Y
75	HCN-NOS	M	16.00	III	HCN-NOS	HCN-NOS	Y
76	HB	F	1.08	III	fetal	fetal	Y
77	HCN-NOS	M	13.64	IV	HCN-NOS	HCN-NOS	Y
79	HCC	F	16.44	IV	HCC	HCC	Y
80	HB	F	0.63	II	Epithelial, fetal	fetal	Y
81	HB	M	4.65	II	Crowded fetal, embryonal	embryonal	Y
82	HB	F	3.70	IV	Pleomorphic, HCC-like areas		Y
83	HB	F	1.88	III	epithelial type, embryonal, pleomorphic (poorly differentiated), SCUD, mesenchymal type without teratoid features	mixed	Y
84	HCC	M	13.20	I	HCC, moderately differentiated	HCC	N
85	HB	M	6.59	I	epithelial type, embryonal, fetal (crowded), cholangioblastic and blastemal, focal mesenchymal	mixed	Y
86	HB	M	3.29		Metastatic HB; blastemal with focal mesenchymal present		Y
87	HB	F	1.31	III	mixed epithelial and mesenchymal, fetal pattern (mitotically inactive), mesenchymal without teratoid	fetal	Y
91	HB	F	3.02	II	mixed epithelial and mesenchymal, fetal (mitotically active), embryonal, pleomorphic (poorly differentiated)	mixed	Y
92	HB	F	0.82	II	epithelial type, fetal (mitotically active), blastemal, with cholangioblastic differentiation		Y
94	HB	F	0.99	IV	epithelial type, fetal pattern, mesenchymal	fetal	Y
96	UES	F	6.64	II	undifferentiated embryonal sarcoma	UES	Y
97	HB	F	2.83	IV	fetal	Fetal	Y
99	UES	F	6.91	II	undifferentiated embryonal sarcoma	UES	Y
105	HCC	M	10.96		moderately differentiated	HCC	N
108	HCN-NOS	M	11.50	III	Pleomorphic fetal of HB overlap with HCN-NOS	fetal	Y
129	HB	M	4.43	II	mixed fetal, crowded fetal and blastemal with focal pleomorphic and cholangioblastic differentiation	mixed	Y
130	HB	M	5.20	I	crowded fetal, embryonal, mixed with blastemal	mixed	Y

Supplemental Table 3- Antibodies and real time PCR primers

Antibodies	Dilution	Company	Product Number	Publication	RRID
IHC/IF					
EZH2	1:200	Cell Signaling	3147	PMID: 35301492	AB_10694383
SUZ12	1:1,000	Cell Signaling	3737	PMID: 36612203	AB_2196850
EED	1:200	Cell Signaling	85322	PMID: 36428492	AB_2923355
H3K27me3	1:1,000	Cell Signaling	9733	PMID: 39731917	AB_2616029
Alexa Fluor 488 goat anti-mouse IgG	1:2,000	Invitrogen	A11001		
Alexa Fluor 555 goat anti-rabbit IgG	1:2,000	Invitrogen	A21428		

Gene Primers	Company	Product Number
EZH2	Qiagen	PPH02880A
SUZ12	Qiagen	PPH17208A
EED	Qiagen	PPH23422
CTNNB1	Qiagen	PPH00643F
Ki67	Qiagen	PPH01024E
GAPDH	Qiagen	PPH00150F
AURKB	Qiagen	PPH21059F
GPC3	Qiagen	PPH11457B
STAT3	Qiagen	PPH00708F
CDH1	Qiagen	PPH00135F
TGFβ	Qiagen	PPH00508A
MYC	Qiagen	PPH00100B
